# A new cave centipede from Croatia, *Eupolybothrus liburnicus* sp. n., with notes on the subgenus Schizopolybothrus Verhoeff, 1934 (Chilopoda, Lithobiomorpha, Lithobiidae)

**DOI:** 10.3897/zookeys.687.13844

**Published:** 2017-08-01

**Authors:** Nesrine Akkari, Ana Komerički, Alexander M. Weigand, Gregory D. Edgecombe, Pavel Stoev

**Affiliations:** 1 Naturhistorisches Museum Wien, Burgring 7, 1010 Wien, Austria; 2 Croatian Biospeleological Society, Zagreb, Croatia; 3 University of Duisburg-Essen, Essen, Germany; 4 Department of Earth Sciences, The Natural History Museum, Cromwell Road, London SW7 5BD, UK; 5 National Museum of Natural History and Pensoft Publishers, Sofia, Bulgaria

**Keywords:** Biospeleology, COI barcoding, *Eupolybothrus*, new species, SEM, Velebit Mountain

## Abstract

A new species of *Eupolybothrus* Verhoeff, 1907 discovered in caves of Velebit Mountain in Croatia is described. *E. liburnicus* **sp. n.** exhibits a few morphological differences from its most similar congeners, all of which are attributed to the subgenus Schizopolybothrus Verhoeff, 1934, and two approaches to species delimitation using the COI barcode region identify it as distinct from the closely allied *E. cavernicolus* Stoev & Komerički, 2013.

*E. spiniger* (Latzel, 1888) is redescribed and a lectotype is designated for it as well as *E. caesar* (Verhoeff, 1899) to stabilize their respective taxonomic status. The subspecies *E. acherontis wardaranus* Verhoeff, 1937, previously suspected to be a synonym of *E. caesar* (Verhoeff, 1899), is redescribed and its taxonomy revised after the study of type material whereas the identity of *E. acherontis* (Verhoeff, 1900) described from a female from southwest Trebinje (Bosnia and Herzegovina) remains unknown. Type material of *E. stygis* (Folkmanova, 1940) is confirmed to be lost and future designation of neotypes from topotypic specimens is necessary to stabilize its taxonomy. The importance of setal arrangement on the intermediate and 14^th^ tergites and the sexual modifications on the male 15^th^ prefemur for species identification is discussed in the light of present findings, and a review of the species of E. (Schizopolybothrus) that display these traits is also provided.

## Introduction

The genus *Eupolybothrus* Verhoeff, 1907 comprises *ca.* 40 valid and doubtful species and subspecies. All species are described from Eastern European and circum-Mediterranean countries, including the largest Mediterranean islands, Corsica, Crete, Cyprus, Sardinia and Sicily (Zapparoli 2002, Stoev et al. 2010, 2013).

Seven subgenera were defined for the genus *Eupolybothrus*, based on a combination of morphological characters but these groupings remain questionable and perhaps do not reflect phylogenetic relationships (see Stoev et al. 2010, 2013). However, they traditionally helped to facilitate species identification with conventional taxonomic methods. Verhoeff (1934) designated the subgenus Schizopolybothrus and assigned *Lithobius caesar* Verhoeff, 1899 as its type species. This subgenus was later re-defined by Jeekel (1967) and extended to include all species with posterior projections on tergites 9, 11 and 13, having spines 15VCa and 15VCm and a simple apical claw on the ultimate legs (Eason 1983). Jeekel (1967) also divided the subgenus into three species groups, based on male gonopods and modifications of the 15^th^ leg but this subdivision was recently rejected by Stoev et al. (2013), who considered groups II and III as invalid due to an erroneous placement of several species. In group III, *E. zeus* (Verhoeff, 1901) and *E. sissi* (Kanellis, 1959) were for instance synonymized with E. (Mesobothrus) transsylvanicus (Latzel, 1882) by Zapparoli (2002) and *E. excellens* (Silvestri, 1894) was wrongly placed together with *E. tabularum* (Verhoeff, 1937) in group II, based only on female characters.

Currently, the subgenus Schizopolybothrus comprises nine species and subspecies, *viz. Eupolybothrusacherontis* (Verhoeff, 1900), *E. acherontis wardaranus* (Verhoeff, 1937), *E. caesar* (Verhoeff, 1899), *E. cavernicolus* Komerički & Stoev, 2013, *E. excellens* (Silvestri, 1894), *E. leostygis* (Verhoeff, 1899), *E. spiniger* (Latzel, 1888), *E. stygis* (Folkmanova, 1940), and *E. tabularum* (Verhoeff, 1937). Some of these are still poorly known and of uncertain taxonomic status. For example, *E. spiniger*, *E. stygis*, *E. acherontis* and *E. acherontis wardaranus* were known only from their original descriptions (see also Stoev 2001a, b, Stoev et al. 2010). While *E. leostygis* was recently re-described (Eason 1983, Stoev et al. 2013), all attempts to find new material of *E. stygis* have failed and the types are now confirmed to be destroyed (Tuf, personal communication). Here, we 1) describe a new species, sp. n., recently collected in caves of Velebit Mountain in Croatia; 2) compare all species of *Schizopolybothrus* focusing on morphological characters that involve modifications of the male prefemur 15 and the intermediate tergite, and supplementing these with the cytochrome *c* oxidase subunit I (COI) gene for species delimitation; 3) examine and re-describe the type material of *E. spiniger* and *E. acherontis wardaranus* housed in Naturhistorisches Museum Wien and the Zoologische Staatssammlung München (ZSM) respectively; 4) designate a lectotype for both *E. spiniger* and *E. caesar*. *E. acherontis*, described by Verhoeff (1900) from a single female specimen and housed in the Senckenberg Naturmuseum Frankfurt am Main, was not accessible to study.

An integrative approach with a combination of morphological and molecular methods is certainly required to better understand the evolutionary history of this group and delineate the number of valid taxa, towards which the present work is a step.

The present work is part of an ongoing revision of the subfamily Ethopolyinae (Stoev et al. 2010, Porco et al. 2011, Komerički et al. 2012, Stoev et al. 2013).

## Materials and methods

### Morphology

All specimens were collected by hand and preserved in 70% or 96% ethanol. The holotype was photographed in situ using a Canon 400D camera with a 65 mm macro objective. Microphotographs were obtained with a Nikon DS-F2.5 camera mounted on a Nikon SMZ25 stereomicroscope using NIS-Elements Microscope Imaging Software with an Extended Depth of Focus (EDF) patch. For scanning electron microscopy, parts of some specimens were cleaned with ultrasound, transferred to 96% ethanol then to acetone, air-dried, mounted on aluminum stubs, coated with Platinum/Palladium and studied in a JEOL JSM-6335F scanning electron microscope. All images were edited in Adobe Photoshop CS6 and assembled in Adobe InDesign CS6. Type material is shared between the Croatian Biospeleological Society – Croatian Natural History Museum (CBSS), The Natural History Museum Denmark – University of Copenhagen (ZMUC), Naturhistorisches Museum Wien (NHMW), and the National Museum of Natural History, Sofia (NMNHS).

Morphological terminology follows Bonato et al. (2010).

### Molecular species delimitation

The standard DNA barcoding locus, the Folmer-fragment of the cytochrome *c* oxidase subunit I (COI) gene was sequenced to delimit *E. liburnicus* sp. n. from other *Eupolybothrus* species. Mid-body legs of four specimens conserved in 70% and 96% ethanol were sent to the Canadian Centre for DNA Barcoding, Guelph, where standard protocols for DNA isolation, PCR and sequencing were performed. The analysed specimens are stored in The Barcode of Life Data System (BOLD) (Ratnasingham and Hebert 2007) project ‘*Eupolybothrus* in Croatia’ (EUCR). Genetic data for the molecular comparison of *Eupolybothrus* species were retrieved from Stoev et al. (2013). All COI-sequences were aligned using the MAFFT-plugin of Geneious 5.4.7 (Kearse et al. 2012) under the G-INS-i option proposed for less than 200 sequences with global homology. Primer sequences at the 5’ and 3’ ends of the alignment were manually trimmed. Molecular species delimitation was performed using two approaches.

Firstly, we used the Automatic Barcode Gap Discovery (ABGD) method of Puillandre et al. (2012). ABGD semi-automatically screens for the presence of a barcoding gap separating within-species (intraspecific) and between-species (interspecific) genetic diversity. We tested several prior combinations of relative gap width (X; ranging from 0.05 – 1.5), minimal intraspecific distance (Pmin; starting at 0.001) and maximal intraspecific distance (Pmax; 0.02 – 0.20). Pmin and Pmax refer to the parameter space where a barcoding gap can be expected, whereas X defines the width of the gap. The Kimura-2-parameter (K2P) model was used for calculating genetic distances in ABGD (Ts/Tv = 2.0).

Secondly, we applied the inverse statistical parsimony (SP) method proposed by Hart and Sunday (2007) for the molecular delimitation of species based on haplotype networks using the program TCS 1.21 (Clement et al. 2000). A connection probability of 95% was set. Conversely, this means that species, i.e. the number of unconnected haplotype networks, are delimitated by 95% statistical confidence. MEGA 6.06 (Tamura et al. 2013) was used for 1) the construction of a Neighbor-Joining (NJ) topology with the K2P substitution model under the pairwise deletion option and for the final visualization of the molecular delimitation results and 2) the calculation of inter- and interspecific K2P-genetic distances for the delineated species.

### Material studied

In addition to recently collected material of a new species listed under its types below, the following material of previously named species was examined:

Eupolybothrus (Schizopolybothrus) acherontis ssp. wardaranus (Verhoeff, 1937): 1 ♂ Reg. Nr. A 200500641; 1 ♀ juv., slide preparation, Reg. Nr. A20030873, “Mazedonien: Skoplje” (ZSM). Eupolybothrus (Schizopolybothrus) caesar (Verhoeff, 1899): 1 ♂, “Greece, Korfu, K. W. Verhoeff leg.”, NHMW 1452, lectotype, designated here. Eupolybothrus (Schizopolybothrus) cavernicolus Komerički & Stoev, 2013: Ad. ♂ Croatia, Knin, NP Krka, cave Miljacka II, 09.II.2013, M. Lukić leg., CBSS – CHP 536, holotype; 1 ♂ Croatia, Knin, NP Krka, cave Miljacka II, 21.X.2012, A. Komerički leg., CBSS – CHP 552 (specimen poorly preserved, damaged), 1 ♂ Croatia, Knin, NP Krka, cave Miljacka II, 10.IX.2015, A. Komerički leg., CBSS – CHP 570. Eupolybothrus (Schizopolybothrus) leostygis (Verhoeff, 1899): Ad. ♂ Croatia, Dubrovnik, Gromača, cave Špilja za Gromačkom vlakom, 29.IV.2004, R. Ozimec & H. Bilandžija leg., CBSS – CHP 406, BOLD ID: EUCR062; ♂ Croatia, Dubrovnik, Gromača, cave Špilja za Gromačkom vlakom, 27.IV.2010, M. Lukić & A. Komerički leg., CBSS – CHP 502, BOLD ID: EUCR017; ♂ Croatia, Dubrovnik, Gromača, cave Špilja za Gromačkom vlakom, 27.IV.2010, M. Lukić & A. Komerički leg., CBSS – CHP 503, BOLD ID: EUCR018. Eupolybothrus (Schizopolybothrus) leostygis patens (Attems, 1935): 2 ♂♂, Greece, “Epirus, Nisista (bei einer Quelle)”, 01.V.1933, Beier M. leg., NHMW 1453, NHMW 8331, syntypes. Eupolybothrus (Schizopolybothrus) spiniger (Latzel, 1888): Ad. ♂, “Bosnien, 1887 J. Karlinski leg.”, NHMW 1463, don. Latzel Nachlass 1919, lectotype designated here; 1 subad. ♂, Bosnien, 1887 J. Karlinski leg., NHMW 8830, don. Latzel Nachlass 1919, paralectotype.

## Results

### Order Lithobiomorpha Pocock, 1895

#### Family Lithobiidae Newport, 1844

##### Subfamily Ethopolyinae Chamberlin, 1915

###### Genus *Eupolybothrus* Verhoeff, 1907

####### Eupolybothrus liburnicus sp. n.

Taxon classificationAnimaliaChilopodaLithobiidae

http://zoobank.org/56310DD5-FB8E-483F-BF93-CE6AF9679CF1

Figs 1 , 2 , 3 , 4 , 5 , 6 , 11H , 12A, C

######## Material.

**Holotype.** Ad. ♂, Croatia, Obrovac, Gornji Čabrići, cave Plitka peć, under rock, 09.II.2013, A. Komerički leg., CBSS – CHP 545.

**Paratypes. Croatia, Southern Velebit**: 2 ♀, Obrovac, Gornji Čabrići, cave Plitka peć, 23.VI.2012, T. Vujnović, A. Komerički & R. Baković leg., CBSS – CHP 538; 6 juv., Obrovac, Gornji Čabrići, cave Plitka peć, 23.VI.2012, A. Komerički leg., CBSS – CHP 541; 2 ♂♂ subadult, Obrovac, Gornji Čabrići, cave Plitka peć, 20.III.2013, K. Miculinić & A. Komerički leg., CBSS – CHP543); 1 ♀, Obrovac, Gornji Čabrići, cave Plitka peć, 23.V.2013, P. Rade & T. Čuković leg., NHMW8409; 1♂, 1 ♀, Obrovac, Gornji Čabrići, cave Plitka peć, 23.V.2012, P. Rade leg., NMNHS CHILOPODA-2016-0003; 1 ♀, Seline, cave Markova špilja, under rock, 01.V.2010, J. Bedek leg., CBSS – CHP 407, BOLD ID: EUCR 048; 1 ♀, Obrovac, Krnjeza canyon, cave Bundalova pećina, 11.VII.2009, R. Baković leg., CBSS – CHP 448; BOLD ID: EUCR 052; 1 ♀, Obrovac, Gornji Čabrići, cave Plitka péc, 29.IX.2009, M. Hodžić leg., CBSS – CHP 457, BOLD ID: EUCR 067; 1 ♂, subadult, Obrovac, Gornji Čabrići, cave Skorupuša, 29.IX.2010, A. Ćukušić leg., CBSS – CHP 458, BOLD ID: EUCR068; 2 ♀♀, 1 juv. Obrovac, Gornji Čabrići, cave Skorupuša, 23.VI.2012, A. Komerički leg., CBSS – CHP539; 7 juv. Obrovac, Gornji Čabrići, cave Skorupuša, 23.VI.2012, A. Komerički leg., CBSS – CHP 540; 1 ♂, 1 juv. Obrovac, Gornji Čabrići, cave Rašeljkovac, 23.VI.2012, A. Komerički leg., CBSS – CHP 544; 1 ♀, Obrovac, Gornji Čabrići, cave Rašeljkovac, 20.III.2013, A. Komerički leg., ZMUC 00040236 ; 1 ♂, Obrovac, Gornji Čabrići, cave Rašeljkovac, 23.IV.2013, K. Miculinić leg., ZMUC 00040237; Mid-body segments and detached legs, Obrovac, Gornji Čabrići, cave Rašeljkovac, 23.V.2013, K. Miculinić leg., CHP: 551, NMNHS CHILOPODA-2016-0001; head and anterior segments, Obrovac, Gornji Čabrići, cave Rašeljkovac, 20.III.2013, A. Komerički leg., CHP: 547, NMNHS CHILOPODA-2016-0002.

######## Diagnosis.

A species morphologically similar to *E. cavernicolus*, genetically differing from it by 11% interspecific distance based on COI, and morphologically differing by the slightly convex posterior margin of T14, presence of 15CxVp and 15PDp spines, and by the leg 15 to body length ratio of *ca.* 64% in the adult male.

######## Description.

**Males.** Based on holotype CHP545 (light photographs) and paratype ZMUC 00040237 (SEM).

**Body length**: (from anterior margin of cephalic plate to posterior margin of telson) approx. 30 mm.

**Figure 1.**
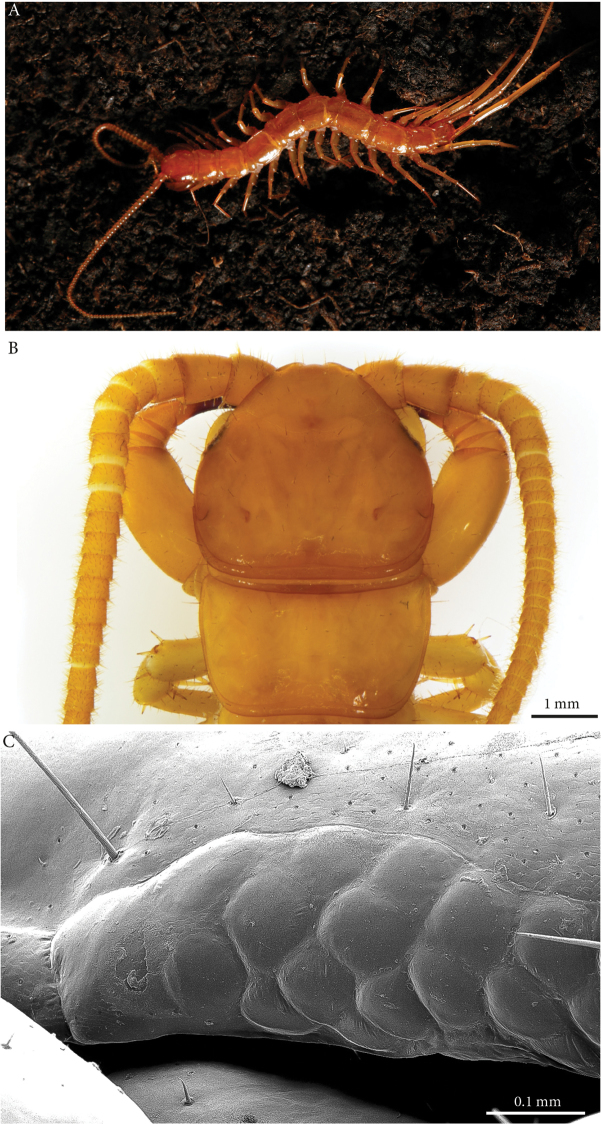
*Eupolybothrus liburnicus* sp. n. habitus, cephalic plate+T1 and ocelli. **A** Habitus, holotype **B** Cephalic plate and T1, holotype, dorsal view **C** Ocelli, paratype ZMUC 00040237.

**Colour**: tawny-brown to yellowish, ventral part and legs paler (Fig. 1A).

**Head**: cephalic plate slightly broader than long (3.6 × 3.1 mm, respectively) and wider than T1 (Fig. 1B); surface smooth, with numerous scattered setae. Cephalic median sulcus contributing to biconvex anterior margin, marginal ridge with a median thickening; posterior margin straight to slightly concave; transverse suture situated at about 1/3^rd^ of anterior edge; posterior limbs of transverse suture visible, connecting basal antennal article with anterior part of the ocellar area.

**Ocelli**: 1+14 blackish irregular ocelli in 3-4 rows; outermost first seriate ocellus largest; ocelli of the middle two rows medium-sized, those of inferior row smallest (Fig. 1C).

**Tömösváry’s organ**: moderately large (as large as a medium ocellus), oval and situated on a sub-triangular sclerotisation below the inferiormost row of seriate ocelli.

**Figure 2.**
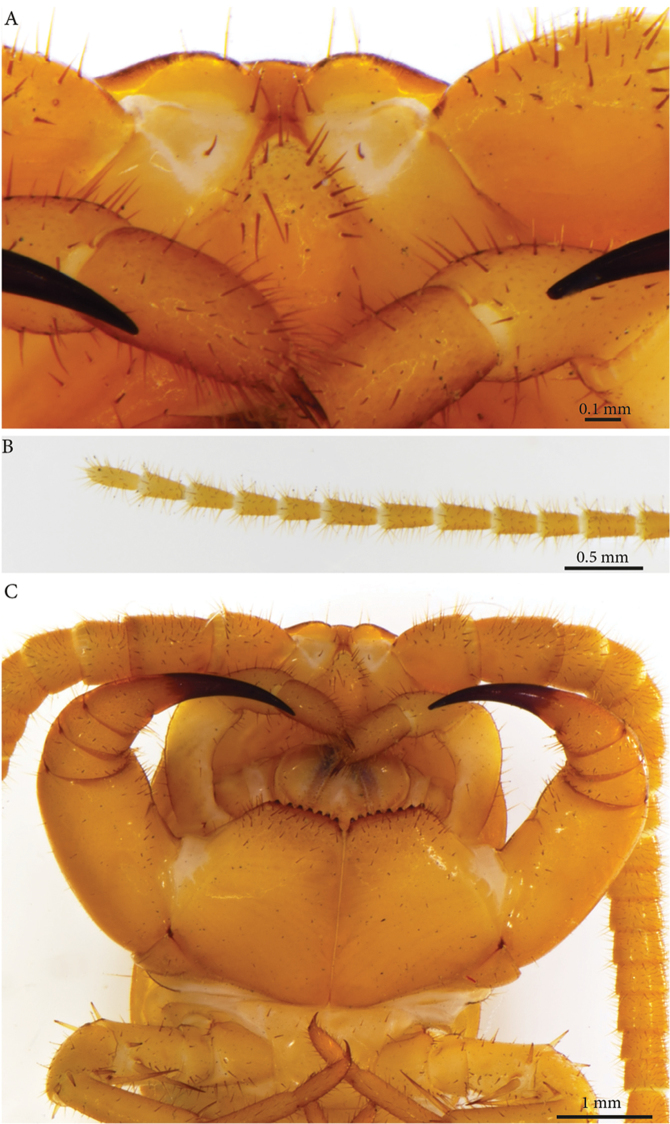
*Eupolybothrus liburnicus* sp. n., holotype head. **A** Clypeus, ventral view **B** Tip of antenna **C** Forcipules, ventral view.

**Clypeus**: showing a cluster of 25 setae situated on the apex and near the lateral margins (Fig. 2A).

**Antennae**: *ca.* 19.8 mm long, ultimate and penultimate articles of same length (Fig. 2B); left antenna composed of 61 articles, right antenna 57 articles; slightly surpassing posterior margin of T7 (right) or T9 (left) when folded backwards, basal two articles enlarged and less setose; the posterior 30 articles visibly longer than broad. Antenna to body length *ca.* 66%.

**Figure 3.**
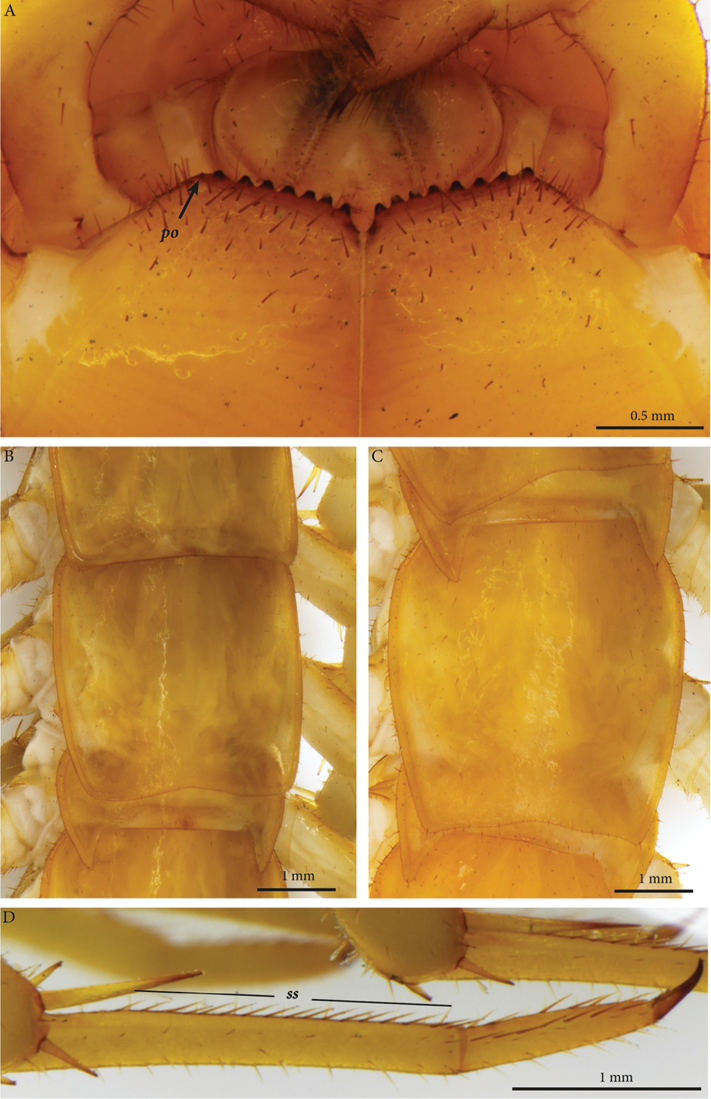
*Eupolybothrus liburnicus* sp. n., holotype, coxosternum, tegites and leg. **A** Close-up of coxosternum, ventral view **B** TT7-9, dorsal view **C** TT11-13, dorsal view **D** Tarsi 1, 2 and pretarsus of midbody leg. Abbreviations: ***po***: porodont, ***ss***: seriate setae.

**Forcipular segment** (Figs 2C, 3A): coxosternite subpentagonal (Fig. 2C), shoulders almost absent (steep), lateral margins straight; anterior margin set off as a rim by furrow; coxosternal teeth 8+7, median diastema small, V-shaped, steep and narrow, short porodont arising from a pit below the dental rim, situated lateral to the lateralmost tooth; base of porodont as thin as adjacent tooth (Fig. 3A, ***po***); coxosternite sparsely setose anteriorly; setae moderately large, irregularly dispersed (Fig. 3A). Forcipular trochanteroprefemur, femur and tibia and proximal part of forcipular tarsungulum with several setae. Distal part of forcipular tarsungulum about 3 times longer than proximal part, *ca.* 2 mm long (Fig. 2C).

**Figure 4.**
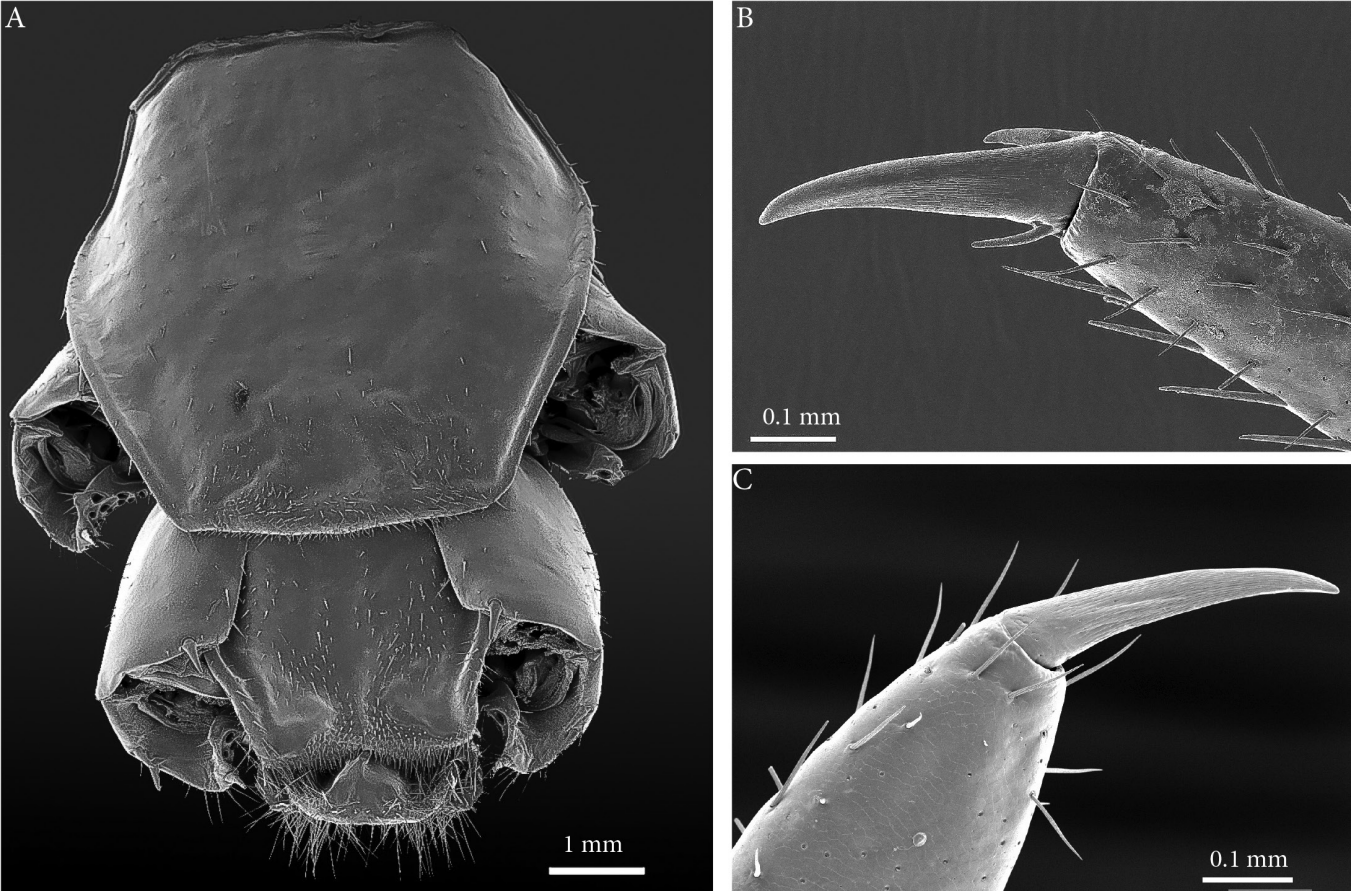
*Eupolybothrus liburnicus* sp. n., male paratype ZMUC 00040237, last tergites and pretarsi of leg 10 and 15. **A** T14 and intermediate tergite, dorsal view **B** Pretarsus of leg 10, dorsolateral view **C** Pretarsus of leg 15, lateral view.

**Figure 5.**
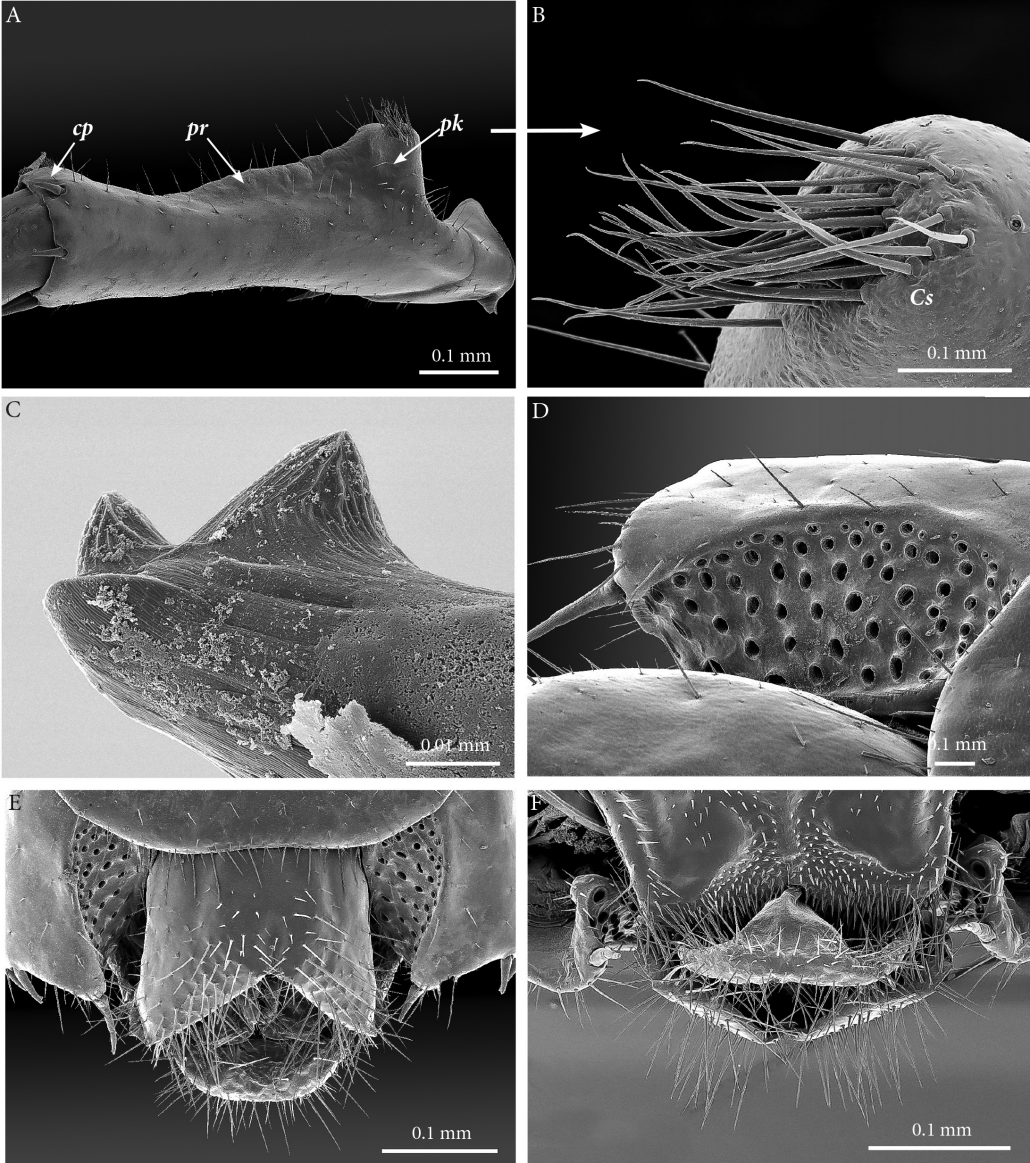
*Eupolybothrus liburnicus* sp. n., male paratype ZMUC 00040237, leg 15 and genital segment. **A** Prefemur 15, posteriodorsal view **B** Close-up of the setae cluster on male prefemur 15 **C** Close-up of the prefemoral spine **D** Coxal pore field, ventral view **E** Genital segment, ventral view **F** Genital segment, dorso-apical view. Abbreviations: ***cp***: circular protuberance, ***pk***: prefemoral knob, ***pr***: prefemoral ridge, ***sc***: cluster of setae.

**Tergites**: T1 wider than long, subtrapeziform, wider anteriorly, posterior margin straight or slightly emarginated, marginal ridge with a small median thickening; TT3 and 5 more elongated than T1, posterior margin slightly emarginated medially, posterior angles rounded; posterior angles of T4 rounded; posterior margin of T8 slightly emarginated medially, angles rounded; TT6 and 7 with posterior angles abruptly rounded (Fig. 3B); TT9, 11, 13 with well-developed posterior triangular projections (Fig. 3B, C); posterior margin of TT10 and 12 distinctly emarginated, that of T14 slightly convex, all strongly setose on posterior margin; intermediate tergite hexagonal, with a membranous area reaching up the 2/3^rd^ of its length (Fig. 11H) and posterior margin straight, lateral edges thickened and covered with setae (Fig. 4A); middle part of posterior third of tergite densely covered with setae (Fig. 5F); laterally, on both sides of the central setose area are two bare subtrapezoidal spots (Fig. 4A). All tergites smooth, setae present only along their lateral margins.

**Legs**: leg 15 *ca.* 64% body length; leg 14 approx. 25% longer than legs 1-12, leg 13 only slightly longer than legs 1-12; pretarsus of legs 1–14 with stouter posterior accessory claw (approx. as long as fundus) and a slightly thinner anterior accessory claw (= spine, sensu Bonato et al. 2010) (Figs 4B, C); pectinal (seriate) setae missing on tarsus1 and 2 of leg 15, present in one short row on tarsus 2 of leg 14, in one row on tarsus 1 and two rows on tarsus 2 of legs 1-13, (Fig. 3D, ***ss***); pretarsus of leg 15 without accessory spines (Fig. 4C). Length of podomeres of leg 15: coxa 1 mm, prefemur 2.7 mm, femur 3.0 mm, tibia 5.3 mm, tarsus 1 4.8 mm, tarsus 2 2.3 mm, pretarsus 0.2 mm. Prefemur of leg 15 with a large proximal knob (Fig. 5A, ***pk***) protruding mediad and bearing a cluster of long setae dorsally (Figs 5B, 11H), in dorsal view the knob as broad as the prefemur, mesally continuing in a ridge reaching 2/3^rd^ of the prefemur length and gently narrowing distad (Fig. 5A, ***pr***). Posterior edge of prefemur with a well-defined circular protuberance densely covered with short, thin setae, situated between *p* and *m* spines dorso-medially (Fig. 5A, ***cp***); rest of prefemur covered with sparse setae. Dorsal spine *p* on prefemur with furcate tip, also noticed on other podomeres and legs (Fig. 5C). Legs 1-14 without particular modifications.

**Coxal pores**: generally round, arranged in 6-7 irregular rows, pores of inner rows largest, size decreasing outwards; pores separated from each other by a distance more than or equal to their diameter; number of pores on leg-pair (measured on right leg) 12: 51, 13: 61, 14: 15: 44 (Fig. 5D).

**Sternites**: smooth, subtrapeziform, with few sparse setae, mainly at lateral margins; posterior margins straight.

**Genitalia**: posterior margin of male first genital sternite concave, posterior margin densely covered with long setae, the rest of sternite sparsely covered with shorter ones (Fig. 5E); gonopod small, concealed above sternite.

**Plectrotaxy**: as in Table 1.

**Figure 6.**
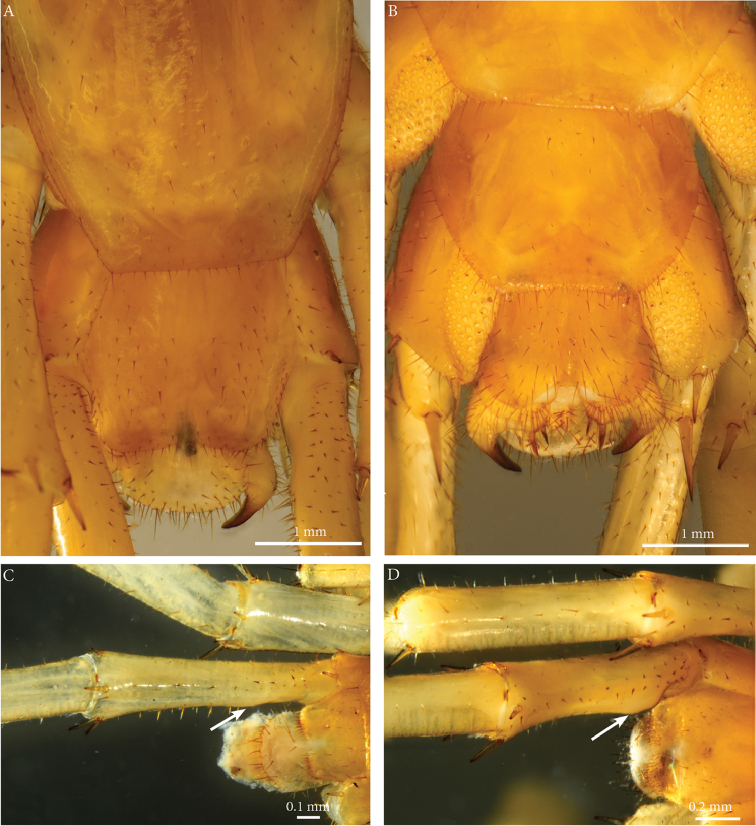
*Eupolybothrus liburnicus* sp. n., A, B, female paratype NHMW 8409. **A** Intermediate and terminal tergite, dorsal view **B** Female terminal sternite and gonopods **C, D** Iimmature males **C** CHP544 **D** CHP543. Arrows point to incipient proximal knob.

**Description of the female, based on paratype NHMW 8409** (Fig. 6A, B).

**Body length**: *ca.* 43 mm; leg 15 *ca.* 22.8 mm or 52.9% body length.

**Colour**: uniformly tawny-brown to yellowish.

**Head**: cephalic plate broader than long (4 × 3.7 mm, respectively).

**Ocelli**: 18 blackish subequal ocelli in 6–7 rows.

**Tömösváry’s organ**: moderately large (as large as or slightly larger than a medium ocellus), oval, situated slightly above the cephalic edge below the inferiormost row of ocelli.

**Clypeus**: with a cluster of *ca.* 25 trichoid setae situated on the apex and lateral margins.

**Antennae**: approx. 18.5 mm long, composed of 73 articles.

**Forcipular segment**: coxosternite subpentagonal, shoulders almost absent, lateral margins straight; anterior margin set off as a rim by furrow; coxosternal teeth 9+8.

**Table 1.** *Eupolybothrus liburnicus* sp. n. plectrotaxy. C – Coxa, Tr – trochanter, P – prefemur, F – femur, T – tibia, a, m, p spines in respectively, anterior, medial and posterior position.

++	Ventral					Dorsal				
	C	Tr	P	F	T	C	Tr	P	F	T
1			amp	amp	amp			amp	a-p	a-p
2			amp	amp	amp			amp	a-p	a-p
3			amp	amp	amp			amp	a-p	a-p
4			amp	amp	amp			amp	a-p	a-p
5			amp	amp	amp			amp	a-p	a-p
6			amp	amp	amp			amp	a-p	a-p
7			amp	amp	amp			amp	a-p	a-p
8			amp	amp	amp	a		amp	a-p	a-p
9			amp	amp	amp	a		amp	a-p	a-p
10			amp	amp	amp	a		amp	a-p	a-p
11			amp	amp	amp	a		amp	a-p	a-p
12		m	amp	amp	amp	a		amp	a-p	a-p
13		m	amp	amp	amp	a		amp	a-p	a-p
14	am	m	amp	am	a	a		amp	p	a-p
15	amp	m	amp	amp	a	a		amp	p	p

**Tergites**: T1 wider than long, subtrapeziform, wider anteriorly, posterior margin straight or slightly emarginated, marginal ridge with a small median thickening; TT3 and 5 more elongated than T1, posterior margin slightly emarginated medially, posterior angles rounded; posterior angles of T4 rounded; posterior margin of T8 slightly emarginated medially, angles rounded; TT6 and 7 with posterior angles abruptly rounded; TT9, 11, 13 with well-developed posterior triangular projections; posterior margin of TT10 and 12 slightly emarginated, that of T14 transverse, all with scarce setae on posterior margin; intermediate tergite hexagonal, posterior margin slightly concave, lateral edges setose, its surface with scattered setae in a few rows located on the lateral margins and the posterior half (Fig. 6A). The rest of the tergites smooth, setae present only on lateral margins.

**Legs**: leg 15 longest *ca.* 22.8 mm, 53% of body length; leg 14 *ca.* 17 mm, leg 13 *ca.* 10.6 mm only slightly longer than legs 1–12, midbody leg (*ca.* 10 mm); pretarsus of legs 1–14 with a more expanded fundus, larger posterior accessory claw (approx. 1/3^rd^ of fundus) and a slightly thinner and shorter anterior accessory claw; pectinal (seriate) setae lacking on tarsi 1 and 2 of leg 15, present in one short row on tarsus 2 of leg 14, and in one row on tarsus 1 and two rows on tarsus 2 of legs 1-13; pretarsus of leg 15 without accessory spines. Leg 15 slender and elongate, without particular modifications.

**Coxal pores**: generally round, forming 6-7 irregular rows, pores of inner rows largest, size decreasing outwards; pores separated from each other by a distance more than or equal to their own diameter (Fig. 6B).

**Sternites**: smooth, subtrapeziform, with few sparse setae, mainly at lateral margins; posterior margins straight.

**Female gonopods**: densely setose, with 2+2 long, slim and pointed spurs slightly bent and a single blunt claw; outer spur 1.5 longer than the inner one, approx. 5 times longer than broad at base (Fig. 6B).

######## Etymology.

*Liburnicus* denotes „of Liburnia“, a district in the coastal region of the northeastern Adriatic; adjective.

######## Variation.

The proximal knob on the male prefemur is substantially smaller in immature males than in mature specimens. For example, CHP544 (body length 11.6 mm) has the prefemoral knob represented by only a low swelling that lacks setae (Fig. 6C), and the medial ridge extending from that swelling is low but distinct; the posterior circular, setose protuberance of adults is indistinct at this size. The posterior part of T14 bears relatively sparse setae, but the tergite of the intermediate segment has a field of dense setae on each side of the midline, and leg 15 is 61% of body length (versus 64% in the holotype). In a male of body length 8.8 mm (CHP543), the prefemur has only a faint bulge in the position of the proximal knob (Fig. 6D), but the tergite of the intermediate segment has a fringe of dense setae on each side of the desclerotized median strip. The female gonopods display spurs with a consistent number (2+2) and sharp, slender shape, with the outer spur on the order of 1.5 times the length of the inner spur. Forcipular teeth are most numerous in the largest specimens, with 6+6 or 7+7 teeth the usual number in specimens less than 25 mm long; some small specimens (e.g., CHP457, body length 10.4 mm) have only 5+5 teeth.

######## Habitat.

*E. liburnicus* sp. n. is here recorded from five caves of the Velebit Mountain, Croatia. Four of these (Plitka peć, Skorupuša, Rašljekovac and Bundalova pećina) are situated in the area where the southern slopes of the Crnopac Massif meet the Krupa River canyon while one of them, Markova špilja, is a small anchialine cave situated a few hundred meters from the Adriatic coast near the village of Seline.

**Figure 7.**
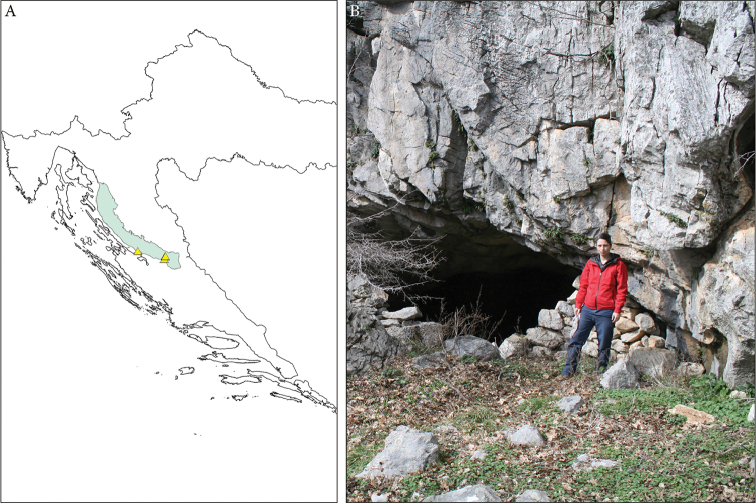
Occurrence of *E. liburnicus* sp. n. in Croatia. **A** Collecting sites (yellow triangle) **B** Entrance of the cave Plitka peć, type locality of *E. liburnicus* sp. n. (photo by K. Miculinić).

The type locality is Plitka peć (Fig. 7), a cave near the village Gornji Čabrići, Obrovac, Zadar County, Croatia. It was formed in Paleogene and Neogene limestone breccias. The cave is small, approx. 30 m long, with a large entrance and a thick layer of sediment on the floor, rich in flowstone and speleothems. The climatic conditions in Plitka peć as measured on 29 September 2010 are as follows: air temperature=12.7° C, sediment temperature=11.4° C, relative humidity=100%. The specimens were collected in both photic and aphotic zones, under stones and in the sediment. The cave is inhabited by spiders of the family Linyphiidae; Pseudoscorpiones: *Neobisium elegans* Beier, 1939, Chthonius (Globbochthonius) sp.; Isopoda: *Alpioniscus* sp., *Androniscus* sp., *Cyphopleon* sp., Collembola: Neelidae, Tullbergiidae, *Lepidocyrtus* sp. (Bregović et al. 2013).

####### Other species

######## Eupolybothrus spiniger

Taxon classificationAnimaliaChilopodaLithobiidae

(Latzel, 1888)

Figs 8 , 11E

Lithobus spiniger Latzel, 1888: 93.

######### Material.

**Lectotype.** adult ♂, Bosnia and Herzegovina, 1887, J. Karlinski leg., NHMW 1463, new designation. **Paralectotype**. 1 subadult ♂, Bosnia and Herzegovina, 1887, J. Karlinski leg., NHMW 8330.

######### Original description

**(translated from Latin).** ‘*Robust, slightly punctate to smooth, posteriorly granulate, chestnut to reddish-brown; glossy. Two antennae slightly elongate, with 50-56 articles. Ocelli on each side: 16-19 (1 + 4, 4, 4, 3 - 1 + 4, 5, 5, 3, 1), in 4-5 longitudinal rows. Forcipular coxosternum: with 14-22 short teeth (7 + 7 - 11 + 11). Tergites 9, 11, 13 with posterior pointed projections, 14 with irregular margin, gradually narrowing posteriad in two pointed projections; coxal pores numerous, round, placed in irregular rows. Ultimate legs: elongate and robust with simple claw; spines: 1, 1, 4, 2, 0-1, coxa with 3 spines on lateral margins. In male ultimate legs, third article (femur) with a large protuberance anteriorly, and indented internal margins. Female: 28-35 mm long, 3.5-4 mm broad*.’

######### Descriptive notes based on the lectotype.

Specimen with broken antenna; 15th legs detached, missing terminal articles, left legs 1, 3, 5 and part of the left forcipule missing.

**Body length**: (from anterior margin of cephalic plate to posterior margin of telson) *ca.* 33 mm.

**Colour**: reddish brown, head and first tergite darker.

**Figure 8.**
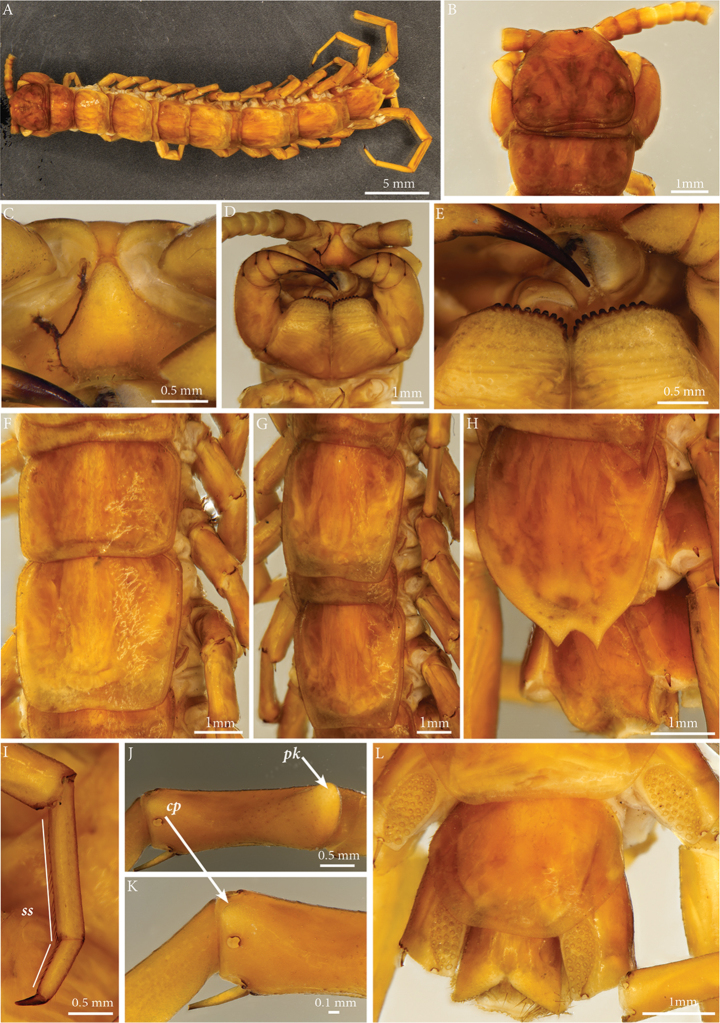
*Eupolybothrus spiniger* Latzel 1889, lectotype NHMW 1463. **A** habitus **B** Cephalic plate, dorsal view **C** Clypeus, ventral view **D** Cephalic plate, ventral view **E** Close-up of the coxosternum, ventral view **F** Tergites 6-8, dorsal view **G** Tergites 9-12, dorsal view **H** Tergites 14-15, dorsal view **I** Tarsi 1, 2 and pretarsus of midbody leg **J** Prefemur of leg 15, ventral view **K** Close-up of the circular protuberance **L** Sternite 14 and intermediate sternite, ventral view. Abbreviations: ***cp***: circular protuberance, ***ss***: seriate setae, ***pk***: prefemoral knob.

**Head**: cephalic plate slightly broader than long (3.5 × 3.8 mm, respectively) and wider than T1 (Fig. 8B); surface smooth, with marks of scattered setae. Cephalic median sulcus contributing to biconvex anterior margin, marginal ridge with a median thickening; posterior margin straight to slightly concave; transverse suture situated at about 1/3^rd^ of anterior edge; posterior limbs of transverse suture visible, connecting basal antennal article with anterior part of the ocellar area.

**Ocelli**: 18, blackish, in 4 irregular rows; outermost first seriate ocellus largest; ocelli of the middle two rows medium-sized, those of inferior row smallest.

**Tömösváry’s organ**: moderately large (as large as a medium ocellus), oval and situated on a sub-triangular sclerotisation below the inferiormost row of seriate ocelli.

**Clypeus**: showing a cluster of 30 setae situated on the apex and near the lateral margins (Fig. 8C).

**Antennae**: Broken, with more than 54 articles.

**Forcipular segment**: Coxosternum with 9+9 teeth and a porodont situated lateral of the distalmost tooth on both sides (Fig. 8D, E).

**Tergites**: T1 wider than long, subtrapeziform, wider anteriorly (Fig. 8B), posterior margin straight or slightly emarginated, marginal ridge with a small median thickening; TT3 and 5 more elongated than T1, posterior margin slightly emarginated medially, posterior angles rounded; posterior angles of T4 rounded; posterior margin of T8 slightly emarginated medially, angles rounded (Fig. 8F); TT6, 7 without posterior projections (Fig. 8G), TT9, 11, 13 with posterior triangular projections (Fig. 8A, H), T14 with posterior margin gradually narrowing into two sub-triangular projections densely covered with setae indicated by marks on tergites (Fig. 8H); intermediate tergite hexagonal, posteriorly emarginated; median part with evident setal marks, laterally with two sub-triangular setae-free spots.

**Legs**: leg 15 18.4 mm long, *ca.* 56% of body length; pectinal (seriate) setae missing on tarsus1 and 2 of leg 15, present in one short row on tarsus 2 of leg 14, in one row on tarsus 1 and two rows on tarsus 2 of legs 1-13 (Fig. 8I, ***ss***). Prefemur of leg 15 with a large proximal knob (Fig. 8J, ***pk***) protruding mediad and possibly bearing a cluster of setae on tip (all setae broken but indicated by marks on prefemur), in dorsal view the knob is less broad than the prefemur and not as round as in *E. caesar* and *E. leostygis*. Mesial ridge thin, reaching 2/3^rd^ the length of the prefemur, gently narrowing distad. Posterior edge of prefemur with a circular protuberance between *p* and *m* dorso-laterally (Fig. 8K, ***cp***); rest of prefemur with obvious marks of setae.

**Coxal pores**: generally round, arranged in 6-7 irregular rows, pores of inner rows largest, size decreasing outwards; pores separated from each other by a distance more than, or equal to their diameter (Fig. 8L).

**Sternites**: smooth, subtrapeziform, with few sparse setae, mainly at lateral margins; posterior margins straight.

**Genitalia**: posterior margin of male first genital sternite concave, broadly V-shaped, posterior margin densely covered with long setae, the rest of sternite sparsely covered with shorter setae (Fig. 8L). Gonopod small, not depicted.

######### Remarks.

*E. spiniger* has not been collected since Latzel’s original description. The type material consists of two syntypes – an adult male and a juvenile - collected in Foča (a town within Republika Srpska, coordinates: 43°30'N, 18°47'E) at approximately 1000 m altitude (Latzel 1888). Stoev et al. (2010) regarded E. (Schizopolybothrus) spiniger as a species of uncertain taxonomic status, presuming it to be a possible senior synonym of *E. caesar* (Verhoeff, 1899), and emphasizing the importance of the examination of the type material. Having now the opportunity to examine the types of *E. spiniger*, we were able to compare it directly with *E. caesar* and conclude that the species is valid, differing from *E. caesar* in several morphological traits, notably the distinctive sub-triangular projections on tergite 14 (see Table 2, Fig. 8H).

**Table 2.** Comparison of standard taxonomic characters in six species of Eupolybothrus subgenus Schizopolybothrus.

			*E. liburnicus* sp. n.	*E. leostygis*	*E. caesar*	*L. spiniger*	*E. cavernicolus*	*E. wardaranus*
			Holotype			Lectotype		Syntype male
			(CHP545)			(NHMW1463)		(Nr. A 200500641)
**Body length (mm)**			30	33–40	24.1 – 31,6	33	22.6–30	29.2
**Head**	**Cephalic plate**	L/W (mm)	3.1/3.6	3.3–4.6/3.0–3.8	2.4–3.7/2.4–3.8	3.5/3.8	3.6/4.0	2/2.5
	**Antennae**	Articles L-R	61–57	73–78	51–58	>54	>61–71	80
		Length	19.8	19.8–28.3 (min)	16,5	broken	20,0–24,0	10.4
	**Ocelli**	number	15	6–7	18–22	R 18	1+14	18–20
		rows	3	1–2	4	4	4	4
	**Coxosternum**	teeth	7+8	9+10 - 11+10	7+8 - 9+9	9+9	8+8	10+11
		setae/side	36	32–48	26–30	broken	22–35	*Ca.* 27
	**Clypeus**	setae	25	20–35	20–21	cca 30	25–30	25
**Coxal pores number/rows**	**12th coxa**	pores/rows	51/6	41–48/5–6	31–34/4–6	44/7	33–36/4–5	62/6–7 (right)
	**13th coxa**	pores/rows	61/6	51/6–7	43–56/5–6	53/7–8	41–44/4–5	55/75–6 (right)
	**14th coxa**	pores/rows	67/7	59–72/6–7	52–70/5–6	60/7–8	49–52/4–5	74/6–7 (right)
	**15th coxa**	pores/rows	44/6	49–71/5–6	40–48/5–6	37/6	34–39/4–5	55/5–6 (right)
**Ultimate legs**	**Coxa**	L/W (mm)	1/0.5	1.0–1.3/0.4–0.5	0.7–1.0/0.3–0.5	1/0.5	1.0–1.5/0.3–0.5	1.07/0.5
	**Prefemur**	L/W (mm)	2.7/0.8	3.3–4.3/0.7–0.9	2.3–3.0/0.6–0.8	3.6/0.7	2.4–3.7/0.8	1.87/0.5
	**Femur**	L/W (mm)	3/0.6	5.0–6.4/0.7	2.6–3.7/0.5–0.7	3.4/0.7	3.4–4.0	2.1/0.4
	**Tibia**	L/W (mm)	5.3/0.6	6.0–7.8/0.5–0.7	3.0–4.4/0.5–0.7	4.3/0.7	4.3–5.2	2.37/0.3
	**Tarsus 1**	L/W (mm)	4.8/0.5	5.3–7.9/0.3–0.5	2.8–4.3/0.5	3.9/0.4	3.8–5.0	2.69/0.3
	**Tarsus 2**	L/W (mm)	2.3/0.4	3.2–4.8/0.2–0.4	2.4–2.8–0.4	1.9/0.3	2.4–3.0	1.57/0.2
	**Pretarsus**	L/W (mm)	0.2	0.4–0.6	0.3–0.4	0.3	0.25–0.4	0.3
**Antenna/body (%)**			*Ca.* 66	>70	*Ca.* 52	–	*Ca.* 75–80	*Ca.* 3
**Ultimate leg length**			19.3	23.2 – 33.1	13.6 – 19.6	18.4	22.5	11.9
**Ultimate legs/body length**			*Ca.* 64%	*Ca.* 75%	*Ca.* 58%	*Ca.* 56%	*Ca.* 74%	*Ca.* 37%
**Setae on intermediate tergite**			Concentrated on posterior margin and on sides of median membranous area, two distinct subtriangular bare fields laterally.	Posterior margin completely covered with dense setae extending anteriad	Concentrated on posterior margin, rare setation, without visible bare fields	Present, concentrated on posterior margin	Wide area of posterior margin completely covered with dense setae, two distinct roundish bare fields laterally.	Concentrated on posterior margin, more setose medially

######## Eupolybothrus wardaranus

Taxon classificationAnimaliaChilopodaLithobiidae

(Verhoeff, 1937) stat. nov.

Figs 9 , 11F

Eupolybothrus acherontis wardaranus Verhoeff, 1937: 100.

######### Material.

**Syntypes.** 1 ♂ Reg. Nr. A 200500641; 1 ♀ juv., slide preparation, Reg. Nr. A20030873, “Mazedonien: Skoplje” (ZSM).

######### Descriptive notes based on the syntype ♂.

**Body length**: (from anterior margin of cephalic plate to posterior margin of telson) approx. 29.2 mm.

**Colour**: uniform, yellowish brown.

**Head**: cephalic plate slightly broader than long (2 × 2.5 mm, respectively) and wider than T1; surface smooth, with scattered setae. Cephalic median sulcus contributing to biconvex anterior margin, marginal ridge with a median thickening; posterior margin straight to slightly concave; transverse suture situated at about 1/3^rd^ of anterior edge; posterior limbs of transverse suture visible, connecting basal antennal article with anterior part of the ocellar area.

**Ocelli**: 18–20, pale, in 4 irregular rows; outermost first seriate ocellus largest; ocelli of the middle two rows medium-sized, those of inferior row smallest.

**Tömösváry’s organ**: moderately large (as large as a medium ocellus), oval and situated on a sub-triangular sclerotisation below the inferiormost row of seriate ocelli.

**Clypeus**: showing a cluster of *ca.* 25 setae situated on the apex and near the lateral margins.

**Antennae**: 10.4 mm, with 80 (left) and 79 (right) articles.

**Forcipular segment**: Coxosternum with 11+10 teeth and a porodont situated lateral of the distalmost tooth.

**Figure 9.**
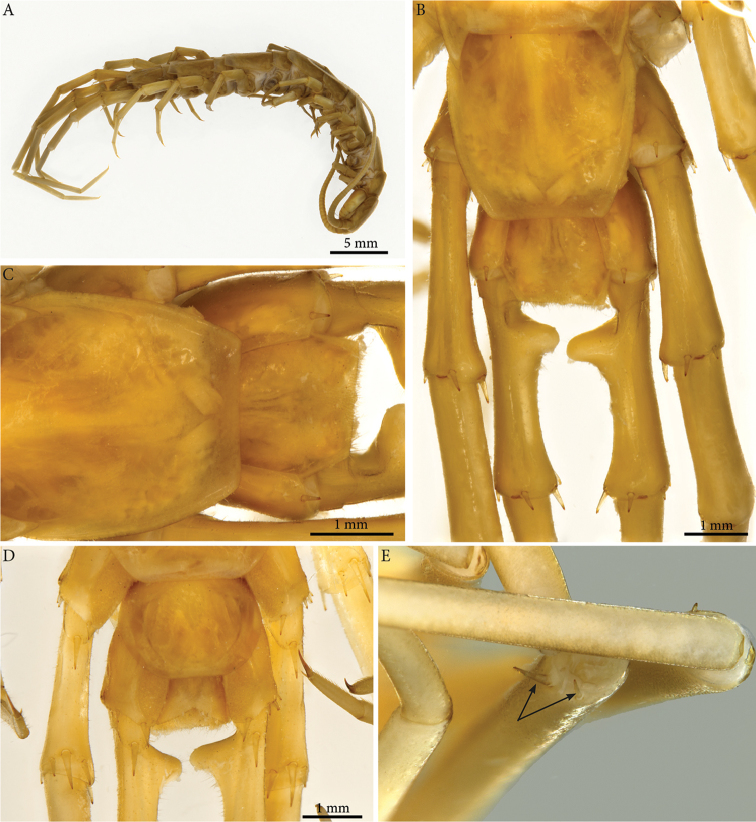
*E. wardaranus*, male syntype. **A** Habitus **B** Terminal part, dorsal view **C** TT14-15, dorsal view **D** Sternites 14-15, ventral view **E** Tibia of leg 15. Arrows pointing to the ventral spines.

**Tergites**: T1 wider than long, subtrapeziform, wider anteriorly, posterior margin slightly emarginated, marginal ridge with a small median thickening; TT3 and 5 more elongated than T1, posterior margin slightly emarginated medially, posterior angles rounded; posterior angles of T4 rounded; posterior margin of T8 slightly emarginated medially, angles rounded; TT6 and 7 with posterior angles abruptly rounded; TT9, 11, 13 with well-developed posterior triangular projections; posterior margin of TT10 and 12 slightly emarginated and 14 almost straight (Fig. 9C); intermediate tergite hexagonal, with a broad median groove narrowing distad and posterior margin almost straight, lateral edges thickened and covered with setae; middle part of posterior third of tergite densely covered with setae; laterally, on both sides of the central setose area there are two specific bare subtrapezoid spots. All tergites smooth, setae present only along their lateral margins.

**Legs**: leg 15 10.9 mm long, *ca.* 37% of body length; pectinal (seriate) setae missing on tarsus 1 and 2 of leg 15, present in one short row on tarsus 2 of leg 14, in one row on tarsus 1 and two rows on tarsus 2 of legs 1-13. Tibia with two ventral spines (Fig. 9E). Prefemur of leg 15 with a large proximal knob (***pk***) protruding mediad and bearing long scattered setae on tip (Fig. 9B). Mesial ridge thin, extending 2/3 of prefemur length, gently narrowing distad. Posterior margin of prefemur without circular protuberance between *p* and *m* dorso-medially; rest of prefemur with obvious marks of setae.

**Coxal pores**: generally round, arranged in 5 irregular rows, pores of inner rows largest, size decreasing outwards; pores separated from each other by a distance more than, or equal to their diameter.

**Sternites**: smooth, subtrapeziform, with few sparse setae, mainly at lateral margins; posterior margins straight.

**Genitalia**: posterior margin of male first genital sternite concave, broadly V-shaped, posterior margin densely covered with long setae, the rest of sternite sparsely covered with shorter setae. Gonopod not depicted.

**Figure 10.**
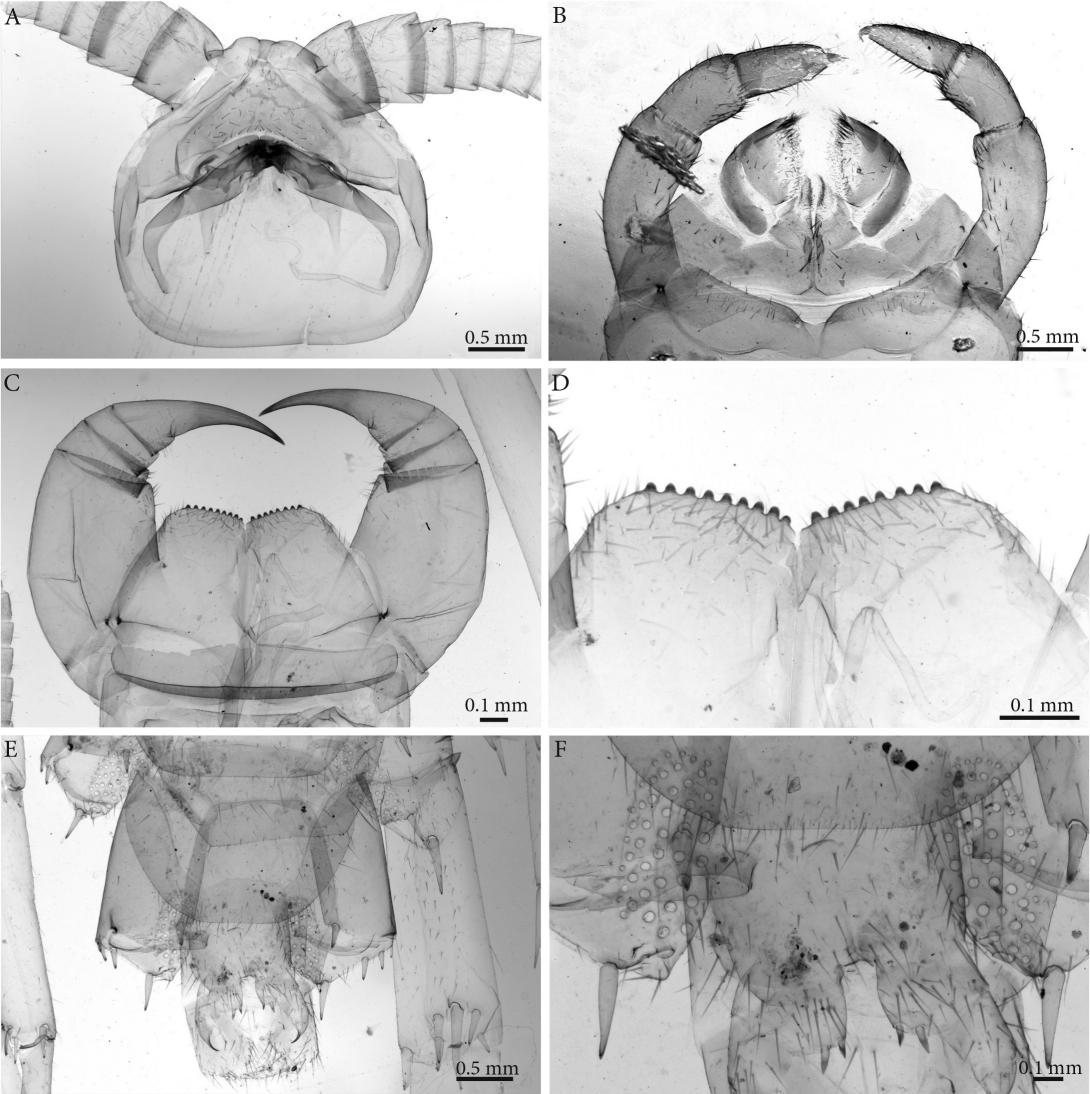
*E. wardaranus*, female syntype. **A** Cephalic plate ventral showing clypeus and mandibles **B** Maxillae **C** Forcipular coxosternum **D** Close-up of the tooth plate **E**, **F** Terminal segments and genitalia, ventral view.

**Description of the syntype** ♀, **based on the slide A20030873 (ZSM)** (Fig. 10).The slide preparation contains the cephalic plate with mandibles *in situ*, maxillae (Fig. 10B), forcipular segment and terminal segments of a female syntype.

**Clypeus**: with a cluster of *ca.* 30 setae situated on the apex, near the lateral margins and smaller one scatted over the surface (Fig. 10A).

**Forcipular segment**: Coxosternum with 8+9 teeth and a porodont situated lateral of the distalmost tooth (Fig. 10C, D).

**Female gonopods**: densely setose, with 2+2 long and pointed spurs slightly bent and a single claw; outer spur 1.5 times longer than the inner one (Fig. 10E, F).

######### Remarks.

**Figure 11.**
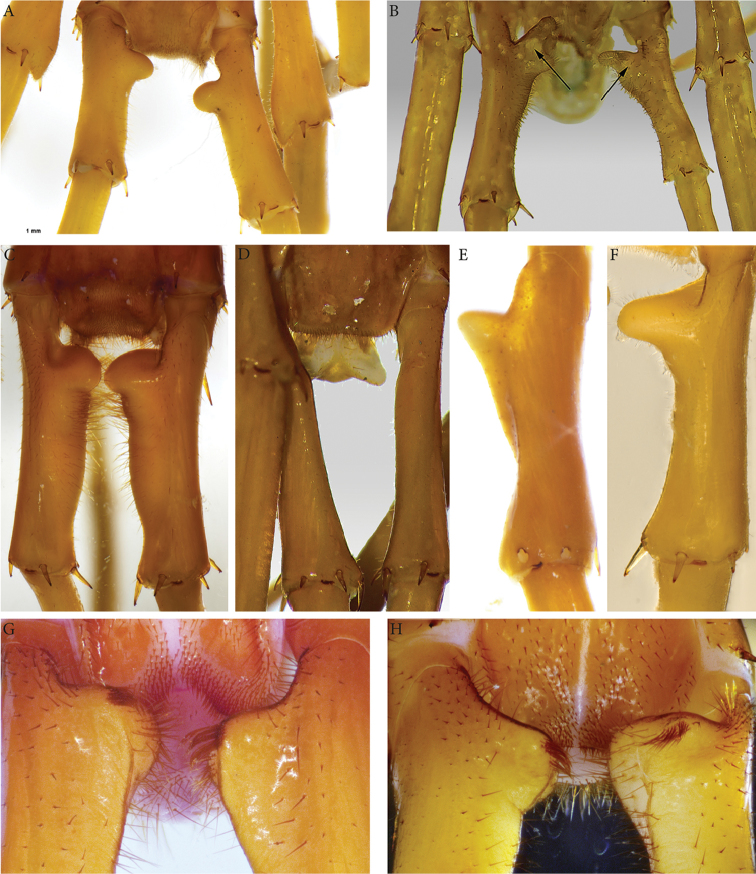
Prefemur of male leg 15 and intermediate tergite, dorsal view. **A** *Eupolybothrus caesar*, syntype **B** *E. excellens* **C** *E. leostygis* **D** *E. tabularum* **E** *E. spiniger*, lectotype **F** *E. wardaranus* **G** *E. cavernicolus*, male holotype **H** *E. liburnicus* sp. n., male holotype. (Figs B and D after Stoev et al. 2010 ZooKeys, https://doi.org/10.3897/zookeys.50.504)

Although originally described as a subspecies of *E. acherontis*, both the nominate subspecies as well as *E. acherontis wardaranus* have subsequently been suspected to be junior synonyms of *E. caesar* (Stoev 2001a, b). Re-examination of its types now shows that *E. wardaranus* can be distinguished from *E. caesar* by the presence of a distomedial projection on the leg 14 prefemur in the latter species (Fig. 11A), versus its absence in *E. wardaranus* (Fig. 9B), and especially by the paired ventral spines on the tibia in *E. wardaranus* (Fig. 9E). *E. caesar* also has a swelling on the dorsal proximalmost part of the leg 15 prefemur (proximal to the knob; Fig. 11A) that is less developed in all other species, including *E. wardaranus* (Fig. 11F). Since *E. acherontis* is known only from a female, the subspecific classification of Verhoeff is difficult to uphold. Accordingly, *E. wardaranus* is treated as a valid species herein.

### Notes on the taxonomy of the subgenus Schizopolybothrus

The taxonomy of the subgenus Schizopolybothrus remains unsolved though it has been recently discussed on two occasions (Stoev et al. 2010, 2013). Among the taxa placed in this subgenus, *E. acherontis* and *E. stygis* remain of uncertain taxonomic status. Except for *E. tabularum* (Fig. 11D), all known members of the subgenus Schizopolybothrus exhibit modifications on the prefemur of ultimate legs in adult males (Fig. 11). The shape and arrangement of setae on the distinct proximal knob of the prefemur, the shape of the ridge on the prefemur, and presence of a circular protuberance with short setae on the distal dorsal part of the prefemur in males are the main distinctive characters for the members of this subgenus. This raises the question whether *E. tabularum* is indeed a member of the same group of species.

### Morphology

Morphological descriptions of species of the genus *Eupolybothrus* have traditionally relied on a number of standard external characters broadly used in lithobiomorph taxonomy (Tobias 1974, Andersson 1981) such as body length, length and width of cephalic plate, number of antennal articles, ocelli, coxosternal teeth, setae on clypeus, coxal pores on legs 12–15, shape of T1 and presence of setae on T14. Providing a valuable overview on the external anatomy of the species, these characters might also be subject to intraspecific variation related to postembryonic development and animal life stage, which renders species identification sometimes impossible or even erroneous when solely relying on them. To address these shortcomings, standard measurements of ratios, also hitherto used in the taxonomy of Lithobiomorpha (Andersson 1981) e.g. antenna and ultimate leg length to body length, as well as length and width of the cephalic plate can be informative for discerning the different taxa (see Table 2).

Focusing on the species attributed to the subgenus Schizopolybothrus, we additionally examined the arrangement of setae on the 14^th^ and intermediate tergite and the shape of its posterior margin in males as well as the presence of lateral setae-free areas (e.g. Figs 11G, H). Males of *E. spiniger* are distinguished by two very noticeable sharp projections on T14 – usually straight in most species – and which were well documented by Latzel (1888) and illustrated here on the type specimen (Fig. 8H).

**Figure 12.**
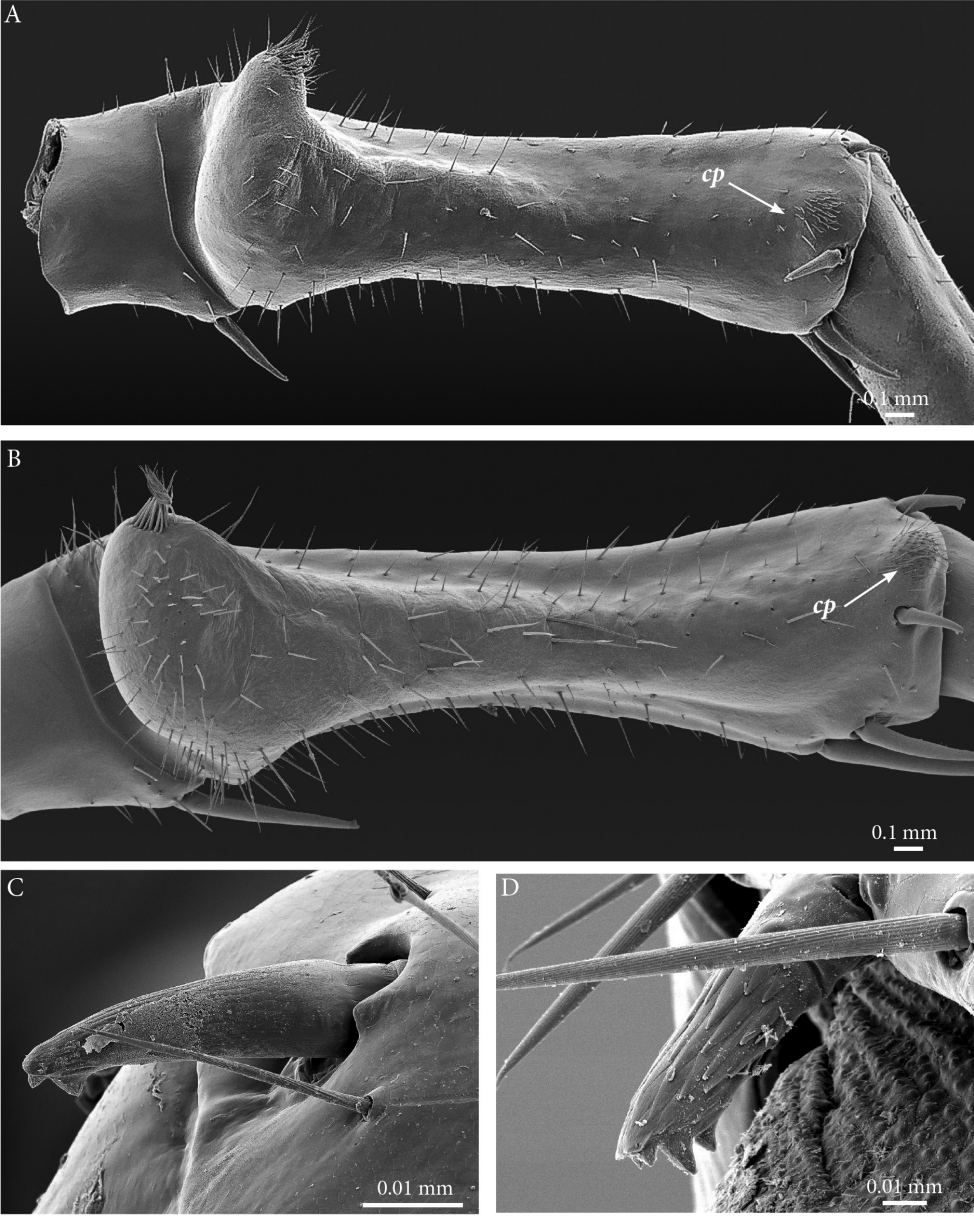
Prefemur of male leg 15 in mediolateral view (**A, B**) and close up of prefemoral spine (**C, D**). **A** *E. liburnicus* sp. n., male paratype **B** *E. cavernicolus*, male paratype **C** *E. liburnicus* sp. n., male paratype ZMUC 00040237 **D** *Eupolybothrus litoralis*. Abbreviation: cp. circular protuberance.

The sexual modification on leg 15 in males in *Schizopolybothrus* was very likely first recorded by Attems (1935) when he described *E. leostygis patens* – a subspecies which is now considered a junior synonym of *E. caesar* (see Zapparoli 1984) – as a setose round protuberance between spines *p* and *m* on the dorsal side of prefemur (“….behaarte Kegel zwischen Dornen p und m.”). In other species of the subgenus Schizopolybothrus, the distal setose circular prefemoral protuberance was detected in *E. caesar*, *E. leostygis*, *E. spiniger*, *E. cavernicolus* and *E. liburnicus* sp. n., situated between the DPm and DPp spines (e.g., Figs 12A, B). It shows subtle variation in size and shape. Further examination of additional species will determine the taxonomic value of this character, as it is also present in congeners that have been classified as other subgenera. For example, Eason (1983: 120, fig. 12) described and figured this structure in *E. herzegowinesis* (confirmed in NHMW1456, Monte Gargano, Apuglia, Italy) and Attems (1902) mentioned it when he described the species *E. werneri* (Attems, 1902) (confirmed in NHMW1430, Parnes, Greece). Verhoeff (1937) described a similar morphology (“… in der Mitte innen angeschwollen und mit Haarbüschel”) in *E. electrinus*, a synonym of *E. imperialis* (Meinert, 1872).

The presence of ‘a knob’ or a swelling on the proximal prefemur of the ultimate legs was first mentioned by Latzel (1888) when he described *E. spiniger* and it was subsequently noted for *E. leostygis, E. caesar, E. excellens, E. cavernicolus* and some other taxa which are now considered their synonyms. The knob extends meso-laterally on the proximal part of the prefemur and exhibits a typical shape for each species. It may be surmounted by a tuft of setae, surrounded by a subapical swirl of setae or covered with scattered ones. An additional feature of the prefemur is also noted, *viz.* ‘a prefemoral ridge’ situated dorso-medially and typically extending distad along two-thirds the length of the prefemur. A comparison of this trait within species of the subgenus Schizopolybothrus is given in Table 3.

**Table 3.** Modifications on male ultimate prefemur in Eupolybothrus species of the subgenus Schizopolybothrus.

	*E. wardaranus*	*E. caesar*	*E. cavernicolus*	*E. excellens*	*E. leostygis*	*E. liburnicus* sp. n.	*E. spiniger*	*E. tabularum*
**Proximal knob**	Subangular, densely setose with thin scattered setae	Round, densely setose (median setae longest) accompanied by a more proximal dorsomedial swelling	Round, protruding mediad, bearing a dorsal tuft of dense setae.	With two protruding densely setose processes	Round, with a subapical whirl of setae (median setae longest)	Round, protruding mediad, bearing a dorsal tuft of dense setae.	Subangular, with probable apical tuft of setae indicated by sockets.	Absent
**Ridge**	Thin, gently narrowing towards the distal third of the prefemur	Even in width, parallel to the prefemur mesal margin	Broad, gently narrowing towards the distal third of the prefemur	Short (less than half length of prefemur), largest proximally, abruptly narrowing	Uniformly broad, narrowing only at the distal third of the prefemur	Broad, gradually narrowing at the distal half of the prefemur	Thin, gently narrowing towards the distal third of the prefemur	Absent
**Circular protuberance**	Absent	Present (small), flat	Present (large), bulged	?	Absent	Present (large), bulged	Present	?

The new species, *Eupolybothrus liburnicus* sp. n., is morphologically and genetically closest to *E. cavernicolus* (Tables 2 and 3). The two species were collected less than 100 km apart in caves with similar but not identical habitat conditions. Morphologically, distinction between these cavernicolous species is subtle but, as discussed below, they are delineated as distinct species by both molecular species delimitation approaches. Morphological distinction is most reliably made using the shape of the posterior margin of T14, plectrotaxy of leg 15, and the length of the ultimate legs relative to the body. In addition, *E. liburnicus* also shows a less setose posterior margin of the intermediate tergite, and usually has a narrower median membranous setae free area on that tergite.

An unusual character depicted and described by Stoev et al. (2013) for *E. cavernicolus* is a bifurcate tip of dorsal spine *p* on the prefemur. This character was further investigated here with SEM and it is also present in *E. liburnicus* (Fig. 12C), *E. leostygis* and *E. litoralis* (Fig. 12D). Whether this character is genus specific or is widely distributed in Lithobiidae remains doubtful and needs to be further examined in other taxa.

### Molecular analyses

**Figure 13.**
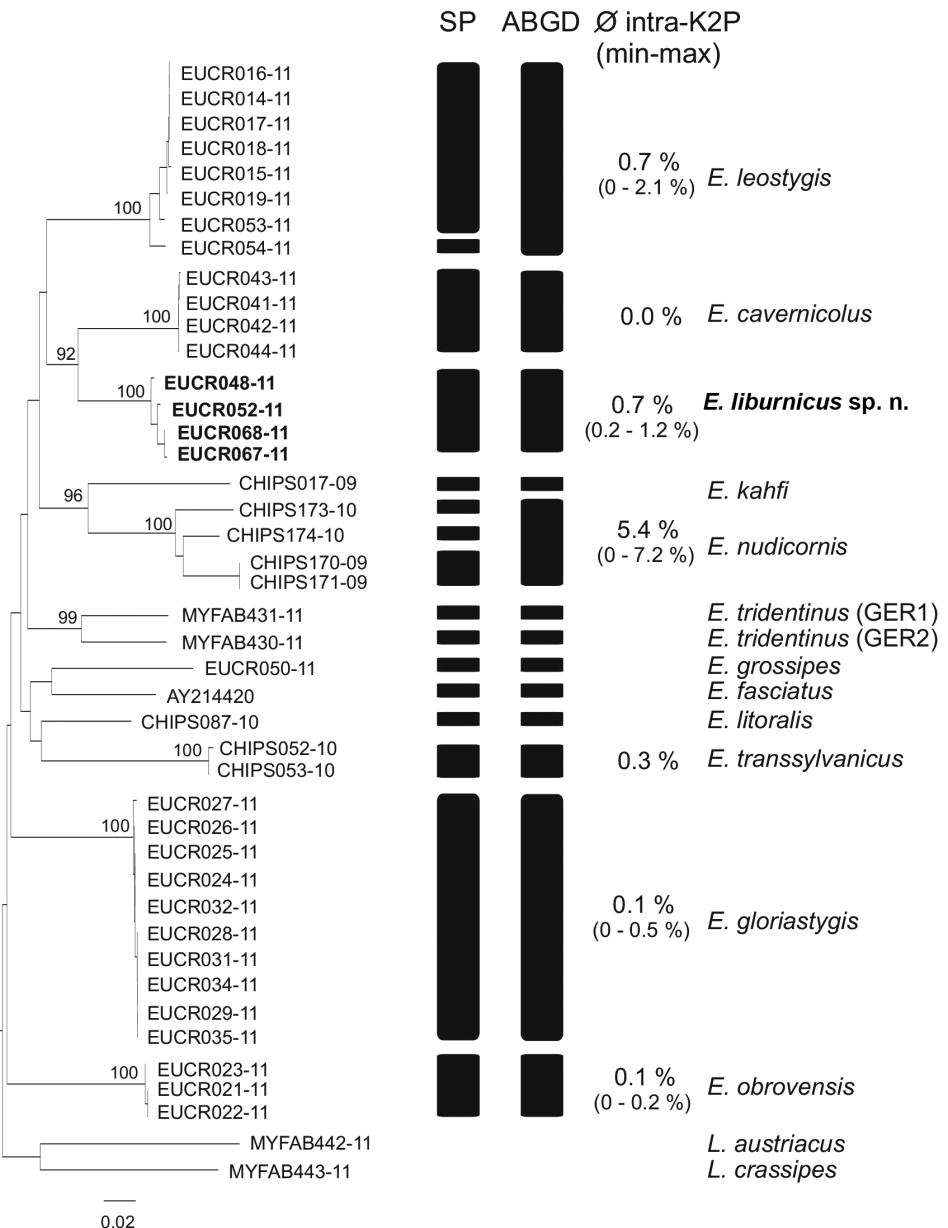
Molecular species delimitation. ABGD proposed 15 clusters congruent to the morphospecies assignments.

The four sequenced specimens of *Eupolybothrus liburnicus* sp. n. provided a full length DNA barcode (BOLD IDs: EUCR048-11, EUCR052-11, EUCR067-11 and EUCR068-11). The final dataset for species delimitation (our four new sequences + sequences of Stoev et al. 2013 and Spelda et al. 2011) consists of 43 specimens representing 12 *Eupolybothrus* morphospecies and two *Lithobius* outgroups: *L. austriacus* (Verhoeff, 1937) (MYFAB442-11) and *L. crassipes* L. Koch, 1962 (MYFAB443-11). Final alignment length was 658 bp with no internal gaps present. Molecular species delimitation via ABGD proposed 15 clusters congruent to the morphospecies assignments (Fig. 13). Only for *E. tridentinus* is a split into two clades proposed. The SP analysis resulted in a total number of 18 clusters, thereby splitting *E. nudicornis* and *E. leostygis* into three and two clusters, respectively. We follow a more conserved approach here and consider the ABGD results for the calculation of inter- and intraspecific genetic K2P-distance values. Nevertheless, specimens of *Eupolybothrus liburnicus* sp. n. always clustered as a single, exclusive group having a mean intraspecific distance of 0.7% (range 0.2–1.2%) and a minimal interspecific distance of 11.0 % to *E. cavernicolus* (Table 4).

**Table 4.** Overview of interspecific K2P-genetic distances of *Eupolybothrus* species.

	*E. gloriastygis*	*E. leostygis*	*E. obrovensis*	*E. cavernicolus*	*E. litoralis*	*E. fasciatus*	*E. tridentinus* GER1	*E. tridentinus* GER2	*E. transsylvanicus*	*E. kahfi*	*E. nudicornis*	*E. grossipes*	*E. liburnicus* sp. n.
***E. gloriastygis*BOLD:AAY5019**													
***E. leostygis*BOLD:AAY5071**	16.7–17.8												
***E. obrovensis*BOLD:AAY5641**	16.2–17.0	18.5–19.4											
***E. cavernicolus*BOLD:AAY4900**	17.6–18.0	14.5–15.4	20.8–21.2										
***E. litoralis***	14.7–15.1	17.1–17.5	17.1–17.3	18.0–18.1									
***E. fasciatus***	16.3–16.8	18.7–19.2	17.5–17.7	21.9–22.1	13.7								
***E. tridentinus* GER1 BOLD:AAV7132**	17.7–18.0	16.7–17.3	18.3–18.5	17.4–17.7	18.0	18.3							
***E. tridentinus* GER2 BOLD:AAV7131**	17.4–17.8	18.6–19.1	19.4–19.7	18.1–18.4	15.7	17.5	10.7						
***E. transsylvanicus*BOLD:AAJ0488**	20.4–21.3	20.7–21.6	21.4–22.1	20.6–20.7	16.0–16.4	20.4–20.8	18.1	19.7–20.1					
***E. kahfi*BOLD:AAY2955**	21.9–22.5	18.9–20.1	21.6–21.8	20.0–20.2	21.0	21.7	22.3	21.5	23.2–23.6				
***E. nudicornis*BOLD:AAN2808 BOLD:AAN2810 BOLD:AAN2811**	20.1–23.2	19.4–21.8	21.1–24.1	21.2–22.7	20.1–21.7	21.7–22.6	20.7–22.4	19.4–21.0	21.4–22.3	17.2–18.8			
***E. grossipes*BOLD:AAY7960**	19.2–19.6	21.0–21.9	20.9–21.1	24.2–24.5	16.6	15.3	20.9	18.9	20.3	22.1	20.7–22.1		
***E. liburnicus* sp. n.**	16.4–17.7	15.0–16.4	19.8–20.6	11.0–11.7	16.6–17.1	18.7–19.3	16.1–16.4	16.6–17.2	20.0–20.5	17.4–17.7	19.5–20.2	20.3–21.0	

Although only three species of E. (Schizopolybothrus) were available for sequencing, they are observed to unite as a monophyletic group (moderate bootstrap support of 65), with *E. leostygis* being sister group to *E. cavernicolus* and *E. liburnicus*. This reconstructed topology would be consistent with a single origin of the prefemoral knob, its associated mesial ridge and the posterior circular setose protuberance in the males of these species.

### Habitat preferences and troglomorphism tendency

Elongation of antennae and legs and reduction of pigment and ocelli are considered morphological adaptations of centipedes to the cave environment (Negrea and Minelli 1994, Voigtländer 2011). In his redescription of *E. leostygis*, Eason (1983) considered long slender forcipules and a slender trunk as further characters of troglobitic species of *Eupolybothrus*. Among the *Schizopolybothrus* species, four were collected solely from caves *viz. E. cavernicolus*, *E. liburnicus* sp. n., *E. stygis* and *E. leostygis*, the last being the only true troglobite. Verhoeff (1900) speculated that *E. leostygis* was actually the cave form of *E. caesar*, and described *E. acherontis* as a transitional form between the two species. Jeekel (1967) argued that the preference to cave habitats by some taxa of *Schizopolybothrus* may result in significant variability in a number of morphological characters and he suggested that the cave dwelling taxa of the subgenus are all cave forms of one troglophilic species.

A number of characters indicative of troglomorphism were noted for the cave dwelling species among those studied herein in contrast to the surface dwelling ones. For instance, *E. caesar* shows the highest number of ocelli (22), the lowest number of antennal articles (51) and setae on coxosternum (26-30), the shortest ultimate leg podomeres (femur, tibia, tarsus 1 respectively 2.6; 3.0; 2.8) and the lowest ratio of ultimate leg to body length (52%). In contrast, *E. leostygis* is characterized by only (6-7) feebly pigmented ocelli (lowest in the subgenus), the longest antenna and ultimate leg podomeres, with the highest ratio of ultimate leg to body length (75%). *E. cavernicolus* and *E. liburnicus* sp. n., display some troglomorphic traits although they are probably not troglobitic, and should be considered as troglophiles. Both species have not been found outside caves although the surrounding areas were thoroughly investigated.

In 2010 and 2012, biospeleological investigations of the caves around Trebinje were conducted by one of us (AK) with the help of cavers from the Caving Club ‘Zelena brda’, which has led to the location of the following: 1) cave Iljina pećina, type locality of *E. stygis* and 2) cave Provalija or ‘Acheron-schlund’ (Verhoeff 1900: 163), type locality of *E. acherontis*. Unfortunately, cave Provalija has turned into an illegal waste disposal site, with its large vertical entrance shaft filled with waste. Iljina pećina has also been destroyed by the opening of an artificial entrance drilled in the 1920s. Both events drastically changed the conditions in these caves and all attempts to find specimens of *Eupolybothrus* there failed. However, recent active sampling was conducted in cave Vučja pećina or ‘Wolfshöhle’ (Verhoeff 1899), the type locality for *E. leostygis*. The cave is fortunately still intact and only *ca.* 300 m from Iljina pećina. In 2012, a single adult female specimen of *Eupolybothrus* was collected by AK, which was of practically no use for this paper. Meticulous sampling in the area would certainly be valuable for future studies on the subgenus, help unveil the identity of some dubious species, and shed some light on the general diversity of the genus *Eupolybothrus*.

### Key to species of Eupolybothrus (Schizopolybothrus) based mainly on male secondary sexual characters

Note. *E. stygis* is based on the description provided by Folkmanova (1940). *E. acherontis* is excluded from the key.

**Table:** 

1	Leg 15 prefemoral knob absent	***E. tabularum***
–	Leg 15 prefemoral knob present	**2**
2	Prefemoral knob apically incised, forming two rounded, densely setose processes	***E. excellens***
–	Prefemoral knob not incised	**3**
3	Posterior edge of T14 deeply emarginated, with sub-triangular posterior processes	***E. spiniger***
–	Posterior edge of T14 slightly emarginated to straight	**4**
4	Prefemoral knob with scattered setae	**5**
–	Prefemoral knob with specific setation (rim or tuft)	**6**
5	Prefemoral knob subangular, projections on leg 14 absent	***E. wardaranus***
–	Prefemoral knob round, projections on leg 14 present	***E. caesar***
6	Prefemoral knob with rim of setae, six poorly defined and feebly pigmented ocelli	***E. leostygis***
–	Prefemoral knob with apical tuft of setae, more than ten pronounced ocelli	**7**
7	10+11 coxosternal teeth, *ca.* 84 antennal articles	***E. stygis***
–	7+7-8+8(9+8) coxosternal teeth, 73 or fewer antennal articles	**8**
8	T14 emarginated, 15CxVp and 15PDp absent	***E. cavernicolus***
–	T14 slightly convex to straight, 15CxVp and 15PDp present	***E. liburnicus***

## Supplementary Material

XML Treatment for Eupolybothrus liburnicus

XML Treatment for Eupolybothrus spiniger

XML Treatment for Eupolybothrus wardaranus
